# Supplementary material for: A novel approach to improving colonoscopy learning efficiency through a colonoscope roaming system: randomized controlled trial
Source: PeerJ Comput Sci. 2023 Jun 9;9:e1409. doi: 10.7717/peerj-cs.1409 (PMC10280502; doi:10.7717/peerj-cs.1409)
Supplement: Supplemental Information 1 [file peerj-cs-09-1409-s001.docx]

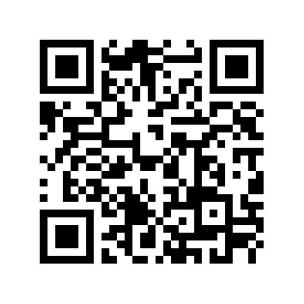


**“Colonoscope Roaming System” Satisfaction Questionnaire**

In order to evaluate the learning efficiency of "Colonoscope Roaming System" software and to improve the software's shortcomings, please give a real and objective feedback after using the GRS, Please mark the selected items. Thank you for your cooperation!

Satisfaction Score: Strongly agree/Agree/Neutral/Disagree/Strongly disagree

| **Number** | **Survey**  **Items** | **Survey Content** | | **Satisfaction Score** | | | | |
| --- | --- | --- | --- | --- | --- | --- | --- | --- |
|  |  |  | | Strongly  agree | Agree | Neutral | Disagree | Strongly  disagree |
| **A** | **Content quality** | Q1 | The e-learning system provides accurate and comprehensive clinical content |  |  |  |  |  |
|  |  | Q2 | The e-learning system provides sufficient content. |  |  |  |  |  |
|  |  | Q3 | The e-learning system provides useful content. |  |  |  |  |  |
|  |  | Q4 | The e-learning system provides content that exactly fits your needs. |  |  |  |  |  |
|  |  | Q5 | The e-learning system provides up-to-date content. |  |  |  |  |  |
| **B** | **Interfac e quality** | Q6 | The e-learning system makes it easy for you to find the content you need. |  |  |  |  |  |
|  |  | Q7 | The e-learning system is user-friendly. |  |  |  |  |  |
|  |  | Q8 | The operation of the e-learning system is stable. |  |  |  |  |  |
|  |  | Q9 | The e-learning system responds to your requests fast enough. |  |  |  |  |  |
|  |  | Q10 | The e-learning system is easy to use and understand. |  |  |  |  |  |
| **C** | **Testing quality** | Q11 | The testing methods provided by the e-learning system are fair. |  |  |  |  |  |
|  |  | Q12 | The e-learning system provides testing results promptly. |  |  |  |  |  |
|  |  | Q13 | The e-learning system makes it easy for you to evaluate your learning  performance. |  |  |  |  |  |
|  |  | Q14 | The e-learning system provides secure testing environments. |  |  |  |  |  |
|  |  | Q15 | The testing methods provided by the e-learning system are easy to  understand. |  |  |  |  |  |

| **D** | **Personal**  **-ization quality** | Q16 | The e-learning system provides the personalized learning support. |  |  |  |  |  |
| --- | --- | --- | --- | --- | --- | --- | --- | --- |
|  |  | Q17 | The e-learning system records your learning progress and performance. |  |  |  |  |  |
|  |  | Q18 | The e-learning system enables you to choose what you want to learn. |  |  |  |  |  |
|  |  | Q19 | The e-learning system enables you to learn the content you need. |  |  |  |  |  |
|  |  | Q20 | The e-learning system enables you to control your learning progress. |  |  |  |  |  |
| **Other comments:** | | | | | | | | |
