# Supplementary material for: A novel approach to improving colonoscopy learning efficiency through a colonoscope roaming system: randomized controlled trial
Source: PeerJ Comput Sci. 2023 Jun 9;9:e1409. doi: 10.7717/peerj-cs.1409 (PMC10280502; doi:10.7717/peerj-cs.1409)
Supplement: Supplemental Information 2 [file peerj-cs-09-1409-s002.docx]

**
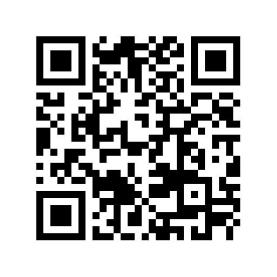
"Colonoscopy Roaming System" Learning Efficiency Test**

（You have 30 minutes to complete the test.）

（The authors:Shizhu Jin and Dandan Ning , Department of Gastroenterology, The Second Affiliated Hospital of Harbin Medical University）

**The examinee name： Scores：**

Single choice questions (each question has only one correct answer. 2 marks for each question, a total of 50 questions, out of 100 marks.)

**1. Which of the following patients can get a colonoscopy? ()**

A. Psychopathic person

B. Severe coagulation disorders

C. The patient refused to sign the informed consent for gastroscopy

D. Patients with severe cardiopulmonary, liver and lung insufficiency

E. Lower gastrointestinal bleeding

**2. During routine colonoscopy, the commonly used position of patients is ()**

A. Left decubitus position with knees bent

B. Right side decubitus position with knees bent

C. Lie flat with knees straight

D. Left lying with knees straight

E. Lie prone with knees straight

**3. As shown in the colonoscopy image, the position of the colonoscopy at this time is approximately ().**


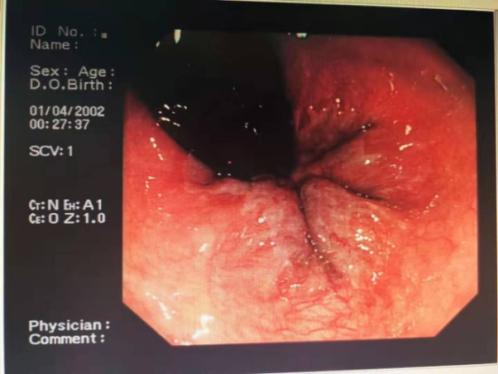


1. Rectum B. Sigmoid colon C. Descending colon D. Transverse colon E. Ascending colon

**4. Which structure of the lower digestive tract is shown in the colonoscopy image ()**


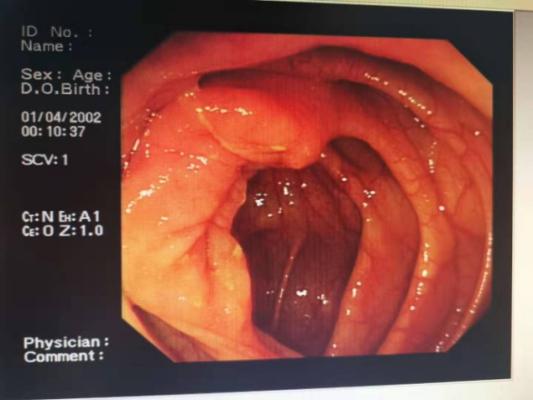


A. liver curvature B. spleen curvature C. anal canal D. appendiceal opening E. ileocecal valve

1. **In normal adults, the distance between liver curvature and anus is about ().**

A. 60 cm B. 50 cm C. 40 cm D. 30 cm E. 20 cm

**6. Which section of the lower digestive tract is shown in the colonoscopic picture ()**


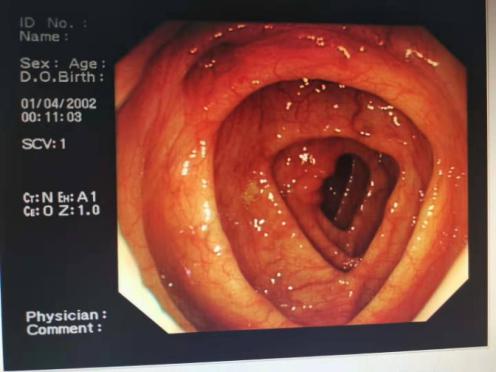


1. Rectum B. Sigmoid colon C. Descending colon D. Transverse colon E. Ascending colon

**7. According to the colonoscopy picture below, it can be diagnosed as ().**


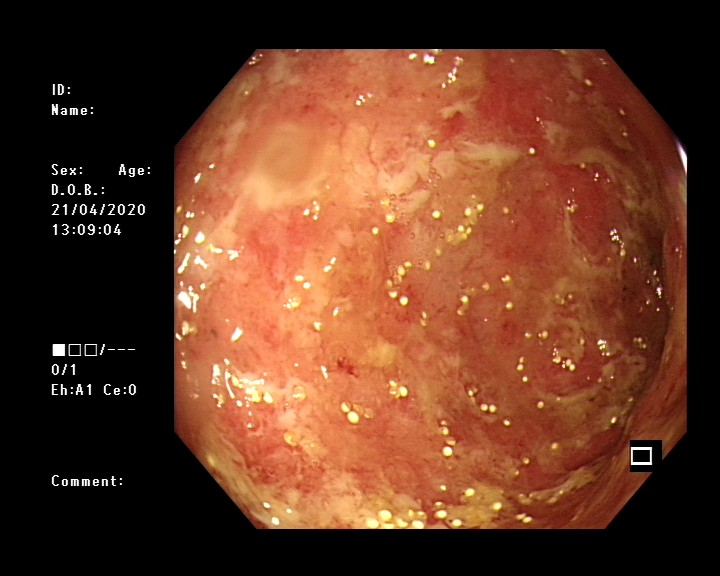


1. Anastomotic stomatitis B. Colonic telangiectasia C. Colon polyps D. Colonic cancer E. Ulcerative colitis

**8. The first colonoscopy after colon cancer surgery should be performed at ()**

A. 6 to 12 months B. 2-3 months C. Over 12 months D. three to six months E. 3-4 months

**9. Which of the following colonoscopic images can be diagnosed as colon polyps?()**

A.
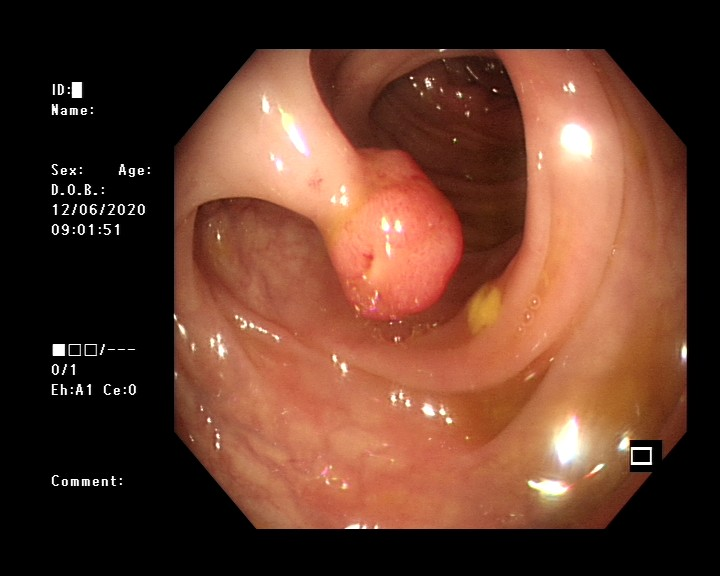
 B.
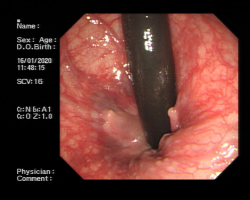
 C.
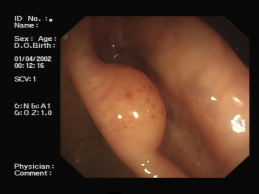
 D.
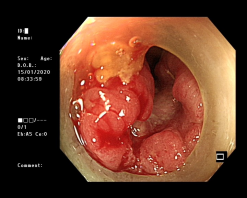


**10. In the colonoscopic pictures below, the colonic diverticulum is ().**

A.
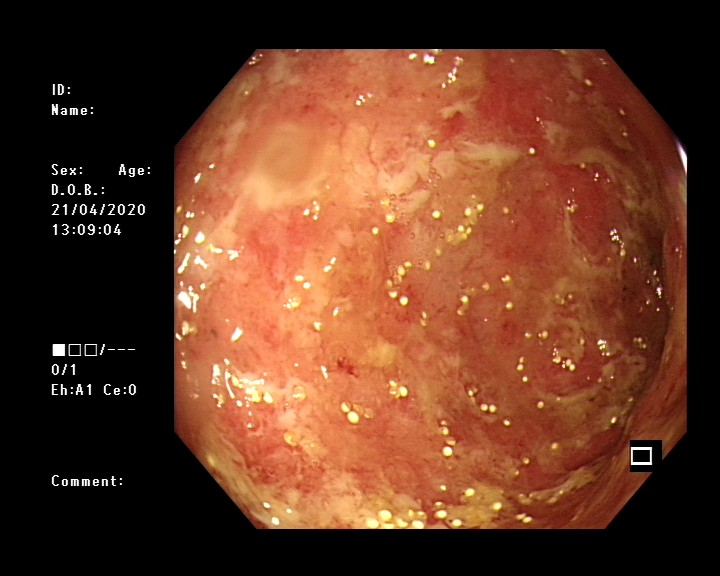
 B.
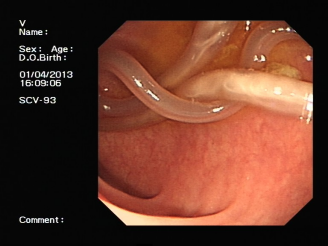
 C.
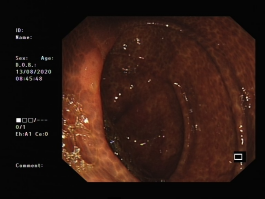
 D.
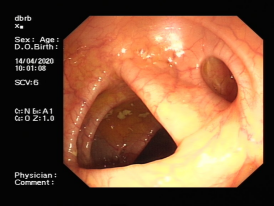


**11. The most serious complication of ulcerative colitis is ()**

A. Colonic pseudo-polyp formation B. Colonic stenosis C. Toxic megacolon

D. Perianorectal abscess E. Lower gastrointestinal bleeding

**12. Which is consistent with the characteristics of pseudomembranous enteritis under endoscopy？**

1.
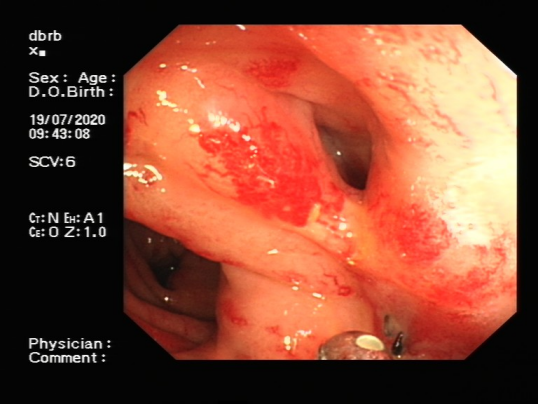
B.
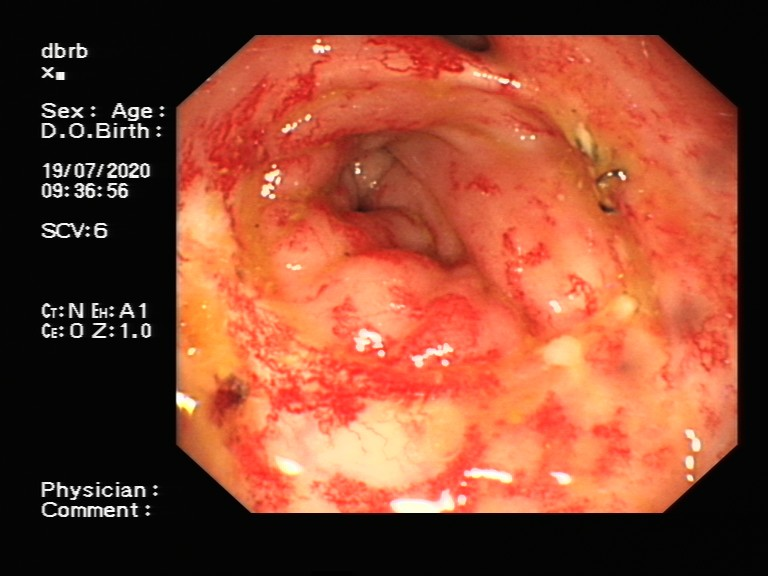


C.
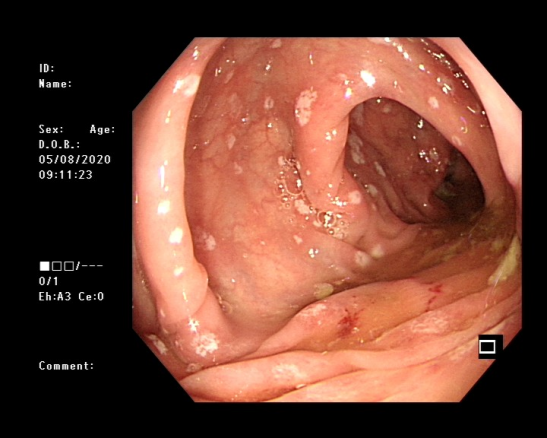
D.
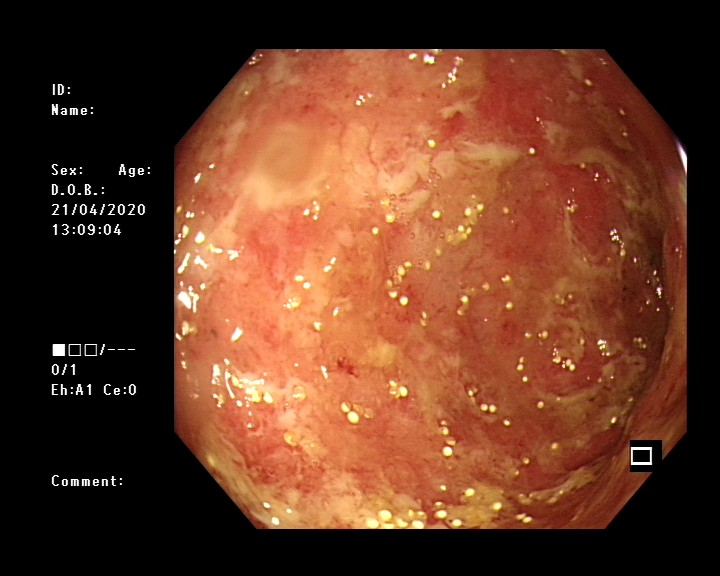


**13. In the following two groups of colonoscopy pictures, which group is the benign ulcer？ ()**


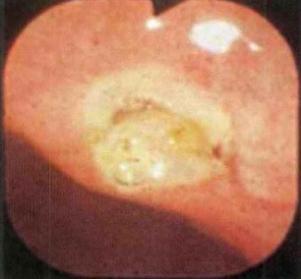
a
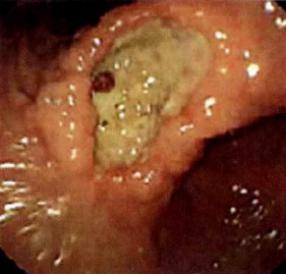
b

A. All benign ulcers

B. A is yes, B is not

C. Picture A is not, Picture B is

D. None of them is benign ulcers

E. Unable to judge

**14. Which endoscopic image can be diagnosed as Yamada type IV？**

1.
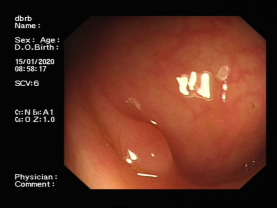
B.
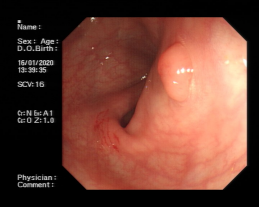
C.
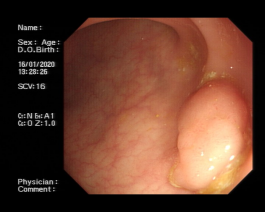
D.
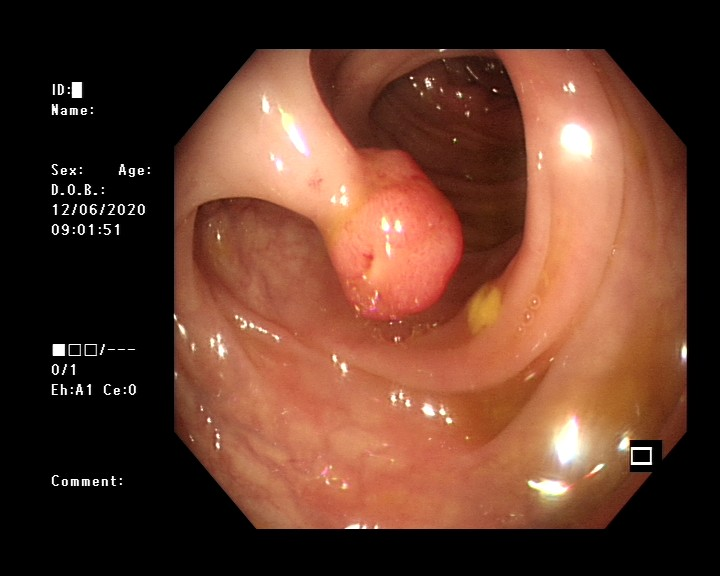


**15. Which is the most common lesion site of Crohn's disease? ()**

A. sigmoid colon B. jejunum C. terminal ileum D. duodenum E. duodenojejunal junction

**16. Male, 25 years old, diarrhea for 5 years, twice a day, accompanied by acute feeling, occasional blood in stool, no fever, stool bacterial culture negative, colonoscopy: sigmoid colon with unclear vascular texture, granular mucosa, easy to bleeding by light touch, the possible diagnose is ()**

A. Intestinal dysfunction B. Moderate Ulcerative colitis C. Mild ulcerative colitis

D. Severe ulcerative colitis E.Crohn's disease

**17. The most common complications of Crohn's disease is ()**

A. Abdominal abscess B. Intestinal obstruction C. Acute perforation D. Urinary calculus E. Spontaneous peritonitis

**18. The colonoscopy picture below can be diagnosed as ().**


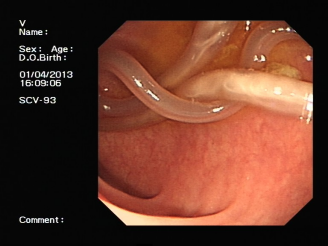


A. Colonic polyps B. Intestinal parasites C. Colonic lipoma D. Colonic leiomyoma E. Colon cancer

**19. The description of the results of the colonoscopy for ulcerative colitis is incorrect.**

A. The mucosa is coarse and fine granular, easy to bleed when touched lightly

B. The ulcers with irregular shapes and different sizes and depths

C. Longitudinal ulcers with normal mucosa between ulcers

D. Inflammatory polyps may be present and colon pouch disappearance

E. Pathology showed crypt inflammation and crypt abscess

**20. What is the possible diagnose of the colonoscopy picture below?**


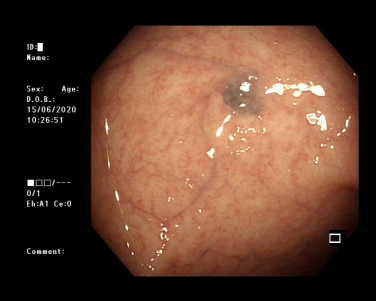


1. Colonic leiomyoma B. Colonic hemangioma C. Colonic lipoma D. Colonic polyp E. Colon cancer
2. **The most commonly used drug for ulcerative colitis is ()**
3. levodopa B.gentamicin C.pipemidic acid D.salazosulfapyridine E.penicillin
4. **Before general endoscopic examination, which of the following statements is wrong?**

A. Verify that there is no fault in the air and water delivery of the endoscope;

B. Hold the operating part of the endoscope in the left hand and adjust the upper and lower, left and right knobs with the thumb;

C. Hold the endoscopic surface with the right hand in pen style, and the holding position is about 25cm away from the end of the endoscope;

D. Apply silicone oil or lubricated painkiller adhesive in front of the lens;

E. Assist the patient in setting up the body position, and tell the patient to relax and cooperate with the examination.

23. Which of the following images conforms to the endoscopic image characteristics of Crohn's? () A.
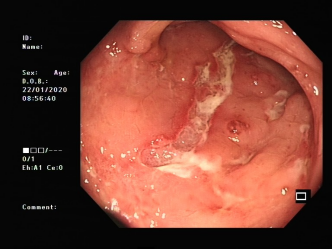
 B.
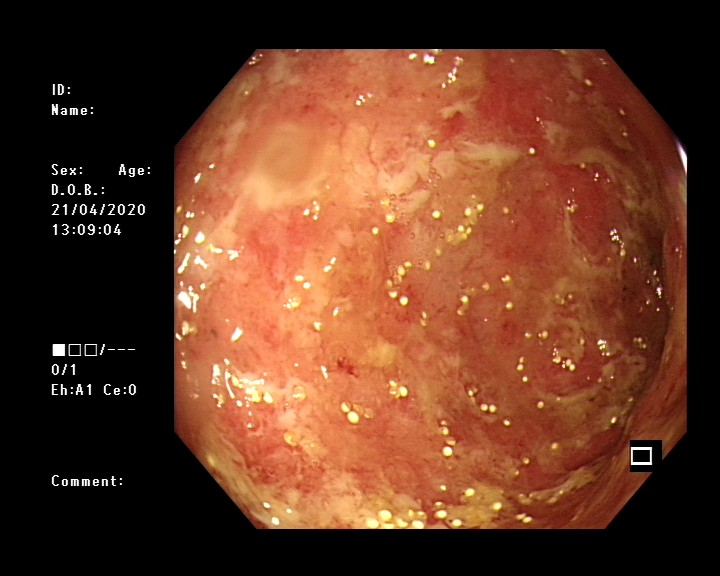


C.
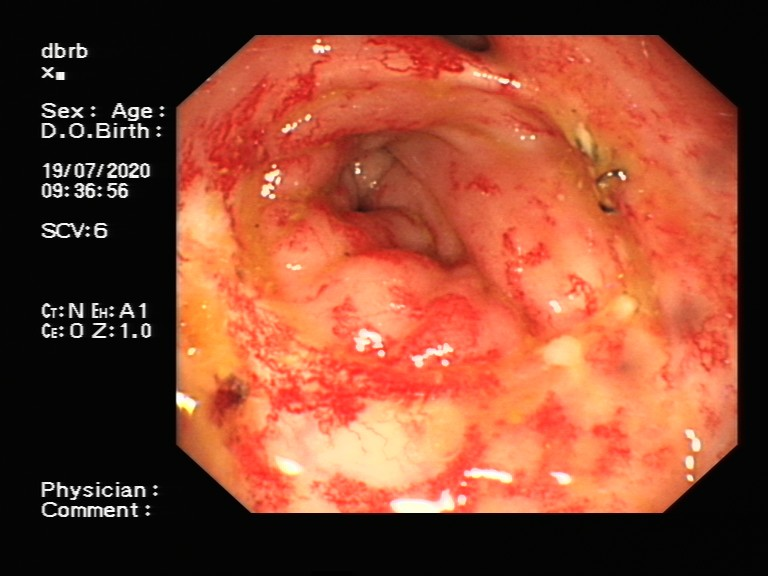
 D.
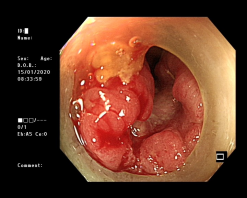


**24. In the following colonoscopy pictures, the person who can be diagnosed with melanosis of the colon is ()**

1.
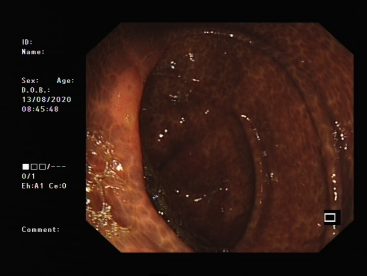
 B.
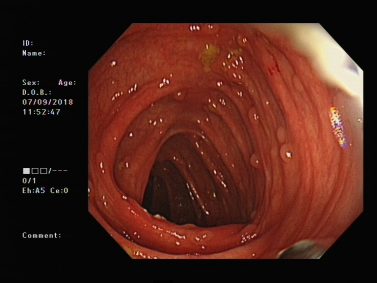


C.
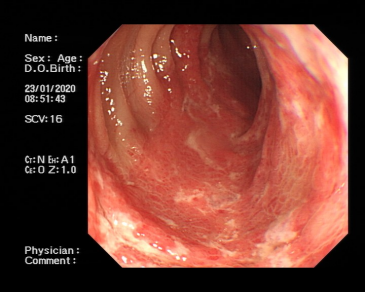
 D.
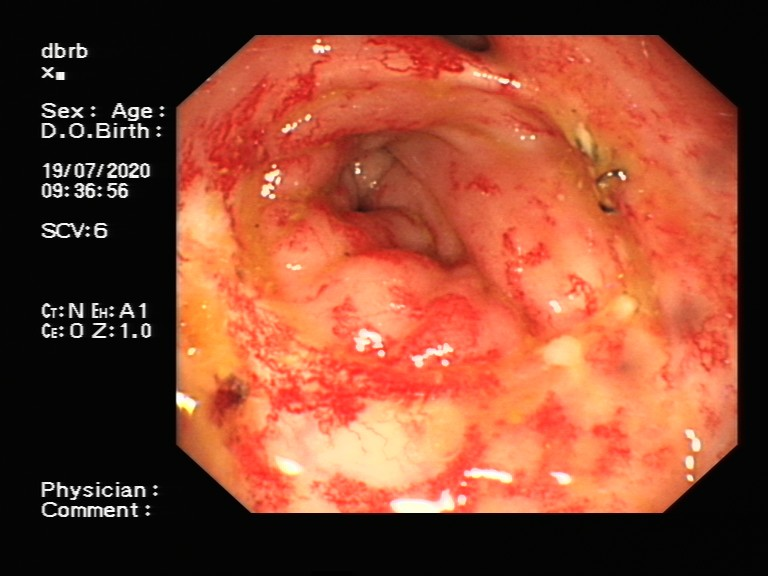


**25. In the following colonoscopy pictures, the one that can be diagnosed as anal papilloma is ()**

1.
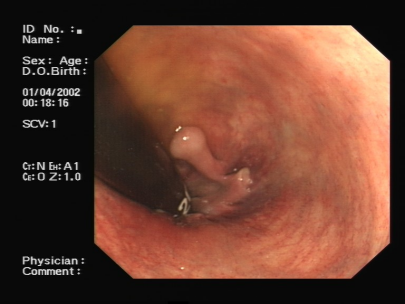
 B.
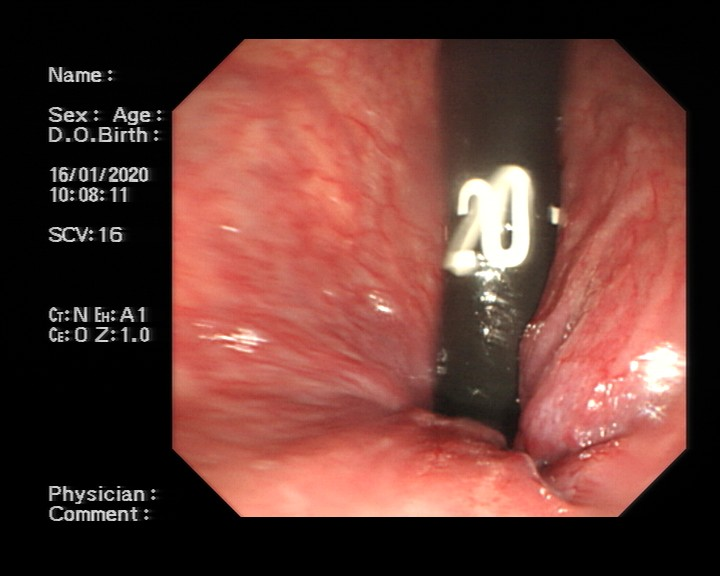


C.
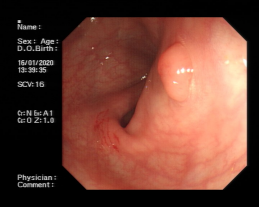
 D.
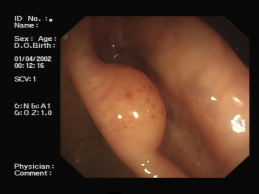


**26. Which statement is false about preoperative preparation for colonoscopy? ()**

A. Start a fluid diet 24 hours before examination

B. After getting up on the day of the examination, absolutely abstain from water and do not take antihypertensive drugs with drinking water

C. Fast four hours before the examination

D. Oral laxative before examination

27. Which statement is not true about the indications for colonoscopy?

A. Lower gastrointestinal bleeding of unknown origin

B. Chronic diarrhea has not been cured for a long time

C. Thin stool

D. Peritonitis and suspected intestinal perforation

28. Which statement is not true about the contraindications to colonoscopy?

A. Severe anal and rectum stricture B. Pregnant women

C. Severe cardiopulmonary failure or mental disorder D. Lower gastrointestinal bleeding

**29. The following colonoscopy image is most likely to be diagnosed as ()**


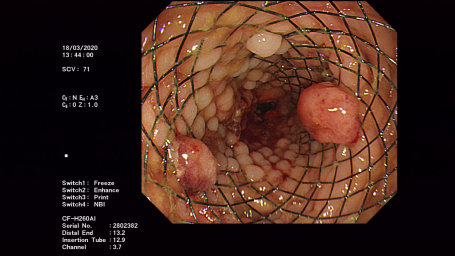


1. Colonic polyps B. Postoperative colon stenting C. Colitis D. Colonic stenosis E. Colon cancer
2. **The following figure can be diagnosed as ()**


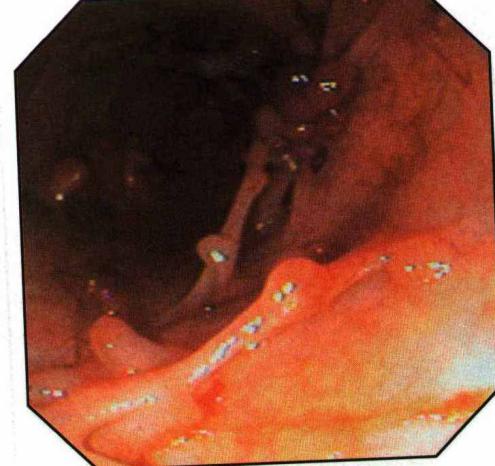


1. Mild ulcerative colitis B. Colon cancer C. Colonic diverticulum
2. Ichaemic bowel disease E. Ulcerative colitis with pseudo-polyp formation
3. **Which image is most likely to be diagnosed as P-J syndrome?**
4.
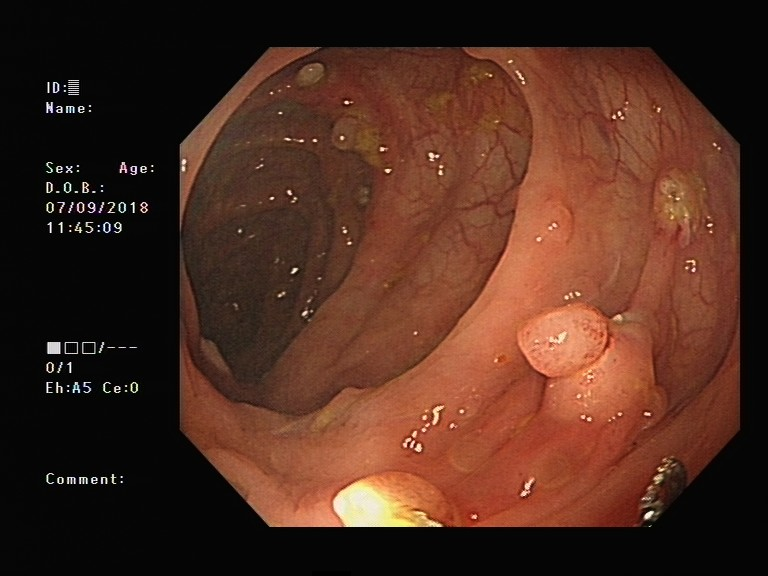
 B.
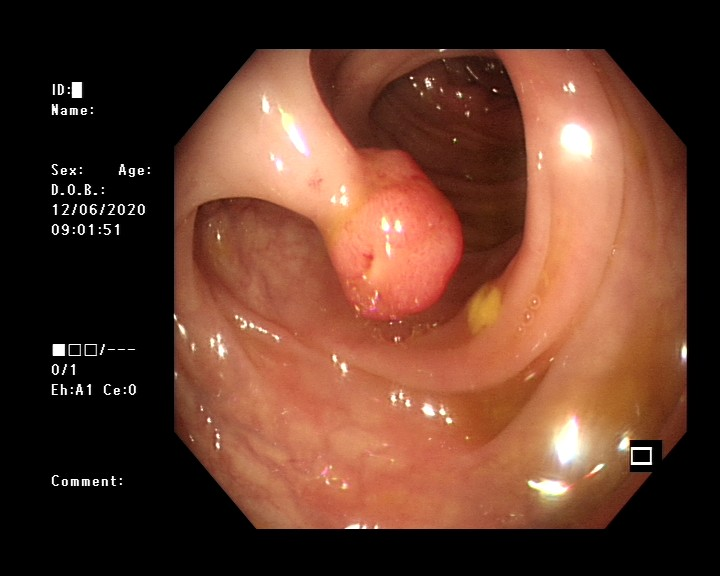


C.
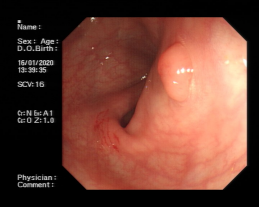
 D.
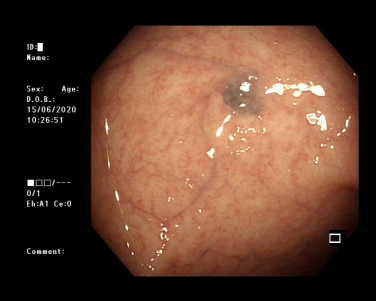


1. **What should be done before colonoscopy ()**

A. No semi-liquid food for 8 hours and no water for 4 hours

B. You should take a laxative to clean the bowel or a cleaning enema

C. Eat enough

D. No smoking, food and medicine for 12 hours and no water for 4 hours

**33. The following figure can be diagnosed as ()**


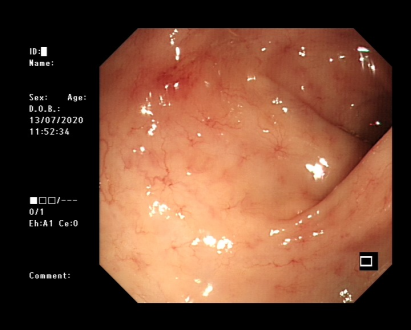


A. Colonic telangiectasia B. Colitis C. Crohn's Disease D. Colon cancer E. Ischemic bowel disease

**34. The following figure can be diagnosed as ()**


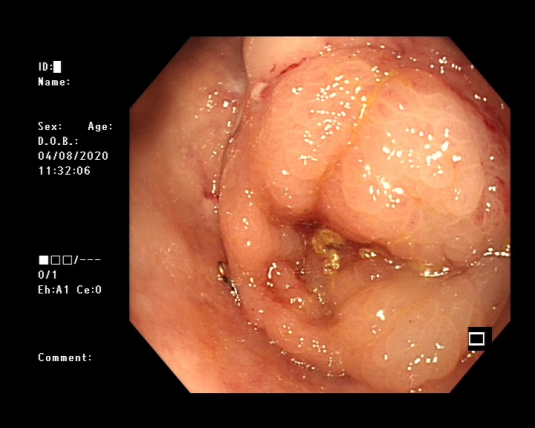


1. Anastomotic stenosis after colonic surgery B. colon cancer C. Colonic polyps

D.Colonic parasites E. Colonic submucosal mas

35. Which of the following colonoscopic pictures is not a colon polyp ()

1.
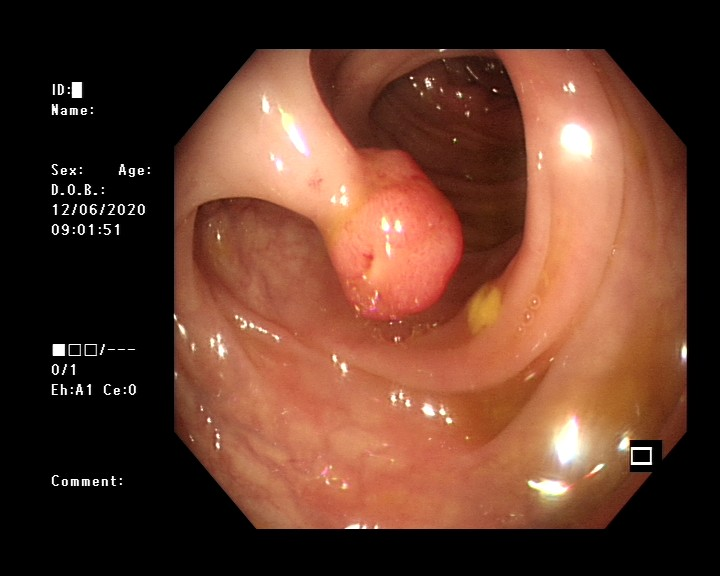
B.
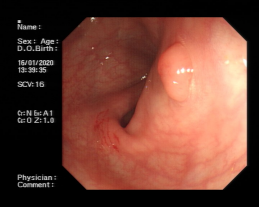
C.
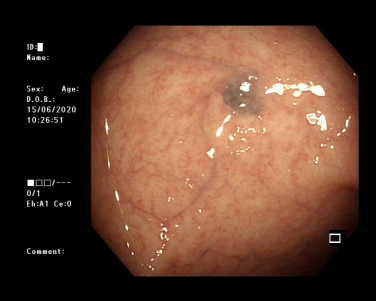
D.
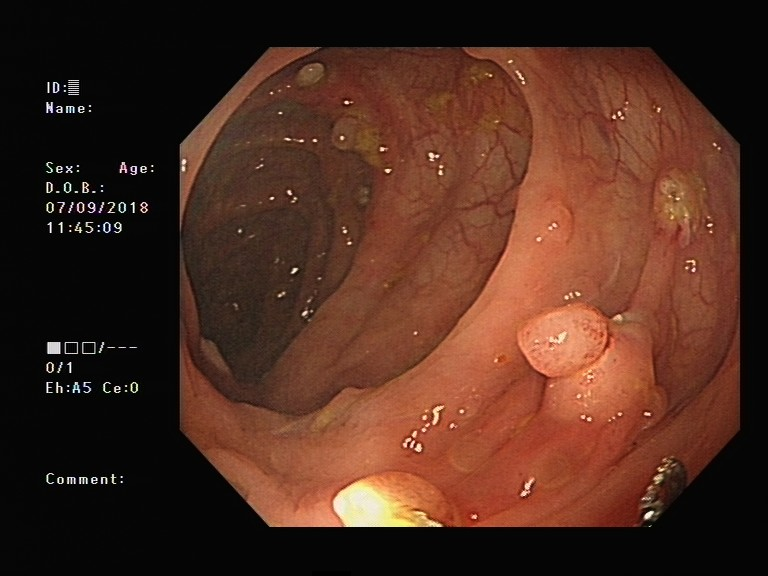


36.What disease can be diagnosed in the picture below?


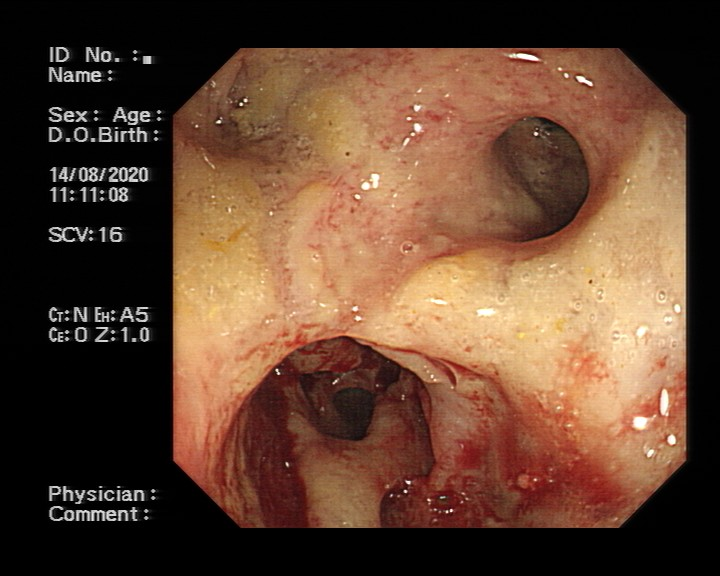


1. Postoperative colonic anastomosis B. Colonic diverticulum C. Colon cancer

D. Colonic parasites E. Colonic polyps

**37. Which is not contraindication of colonoscopy? ()**

A. Acute intestinal infection B. Severe pulmonary insufficiency C. Hematochezia

D. Intestinal adhesions E. Unable to cooperate with the inspector

**38. The following figure can be diagnosed as ()**


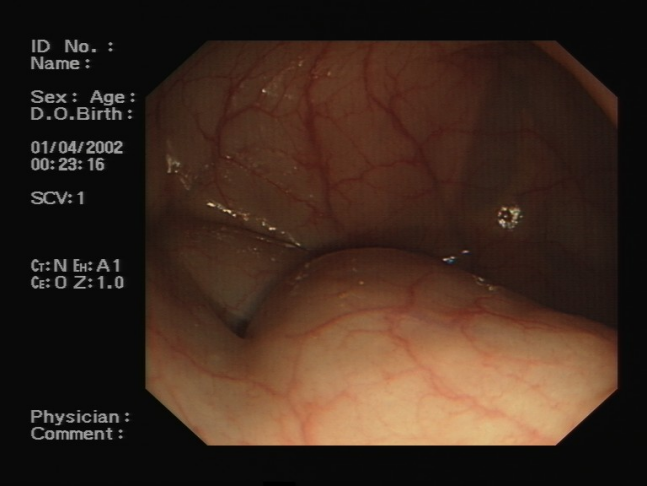


A. External pressure B. Colon cancer C. Colonic polyps D. Colitis E. Ischemic bowel disease

**39. The following figure can be diagnosed as ()**


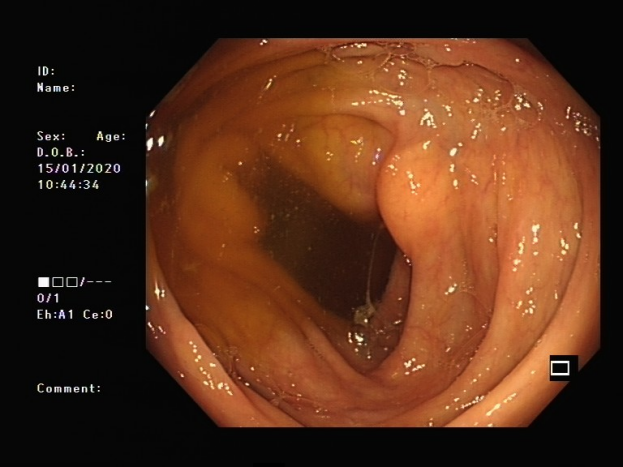


1. Colonic submucosal mass B. Colonic cancer C. Colonic polyp D. Colitis E. Ischemic bowel disease

**40. The following figure can be diagnosed as ()**


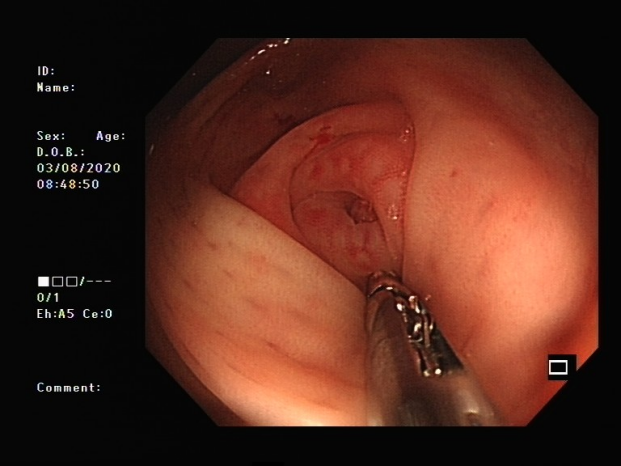


1. Appendicitis B. Ulcerative colitis C. Crohn's disease D. Ischemic bowel disease E. Radiation enteritis
2. **Complications of colonoscopy do not include ()**
3. Intestinal hemorrhage B. Intestinal perforation C. Mesenteric injury

D.Gas explosion E. Cardiovascular and cerebrovascular accident

**42. The following figure can be diagnosed as ()**


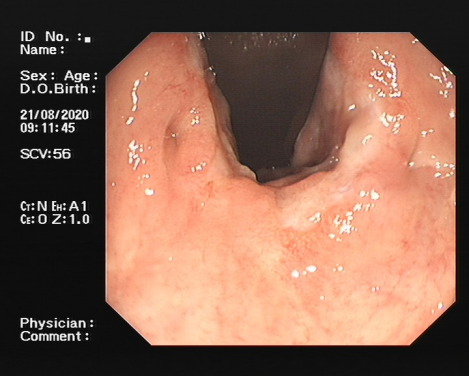


A. anal fistula B. internal hemorrhoids C. anal papilloma D. external hemorrhoids E. mixed hemorrhoids

**43. Which endoscopic examination achieves a high level of disinfection?**

A. Hysteroscopy B. Colonoscopy C. Cystoscope D. All of the above

**44. Which is not terminal ileitis in the picture below?()**

A.
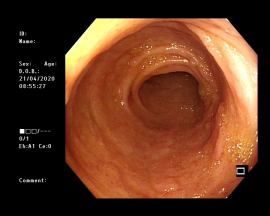
B.
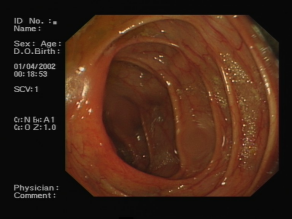
C.
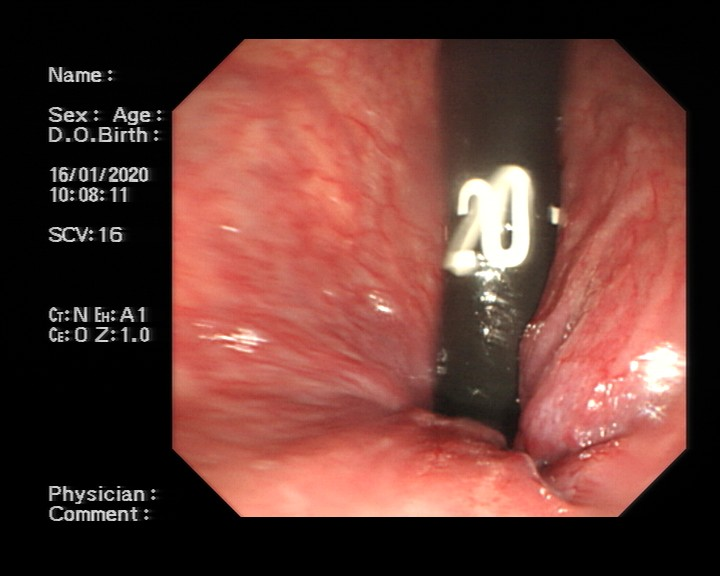
D.
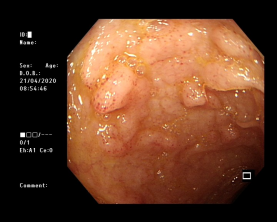


**45. Which of the following images can be diagnosed as lipoma ()**

1.
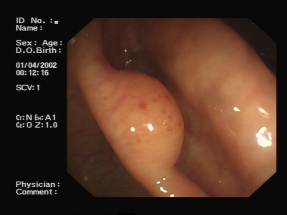
B
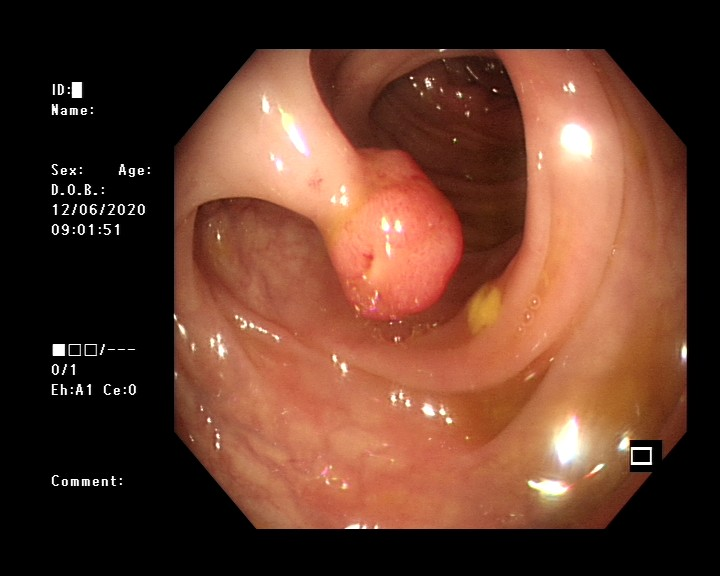
C.
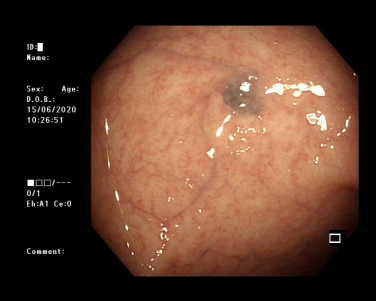
D.
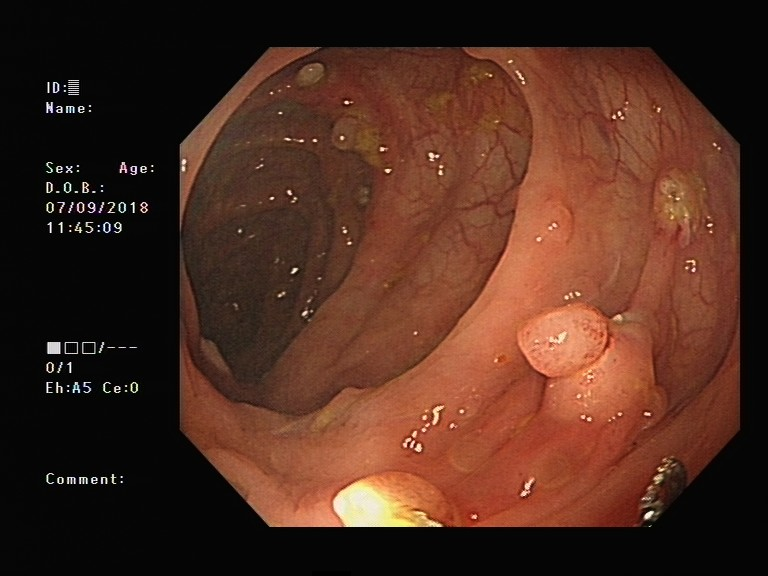


**46. Common site of intestinal tuberculosis is ()**

A. ileocecal B. ascending colon C. transverse colon D. sigmoid colon E. hepatic flexion of colon

**47. Which diagnosis is not ulcerative colitis? ()**

1.
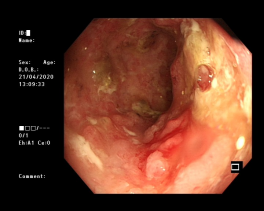
B.
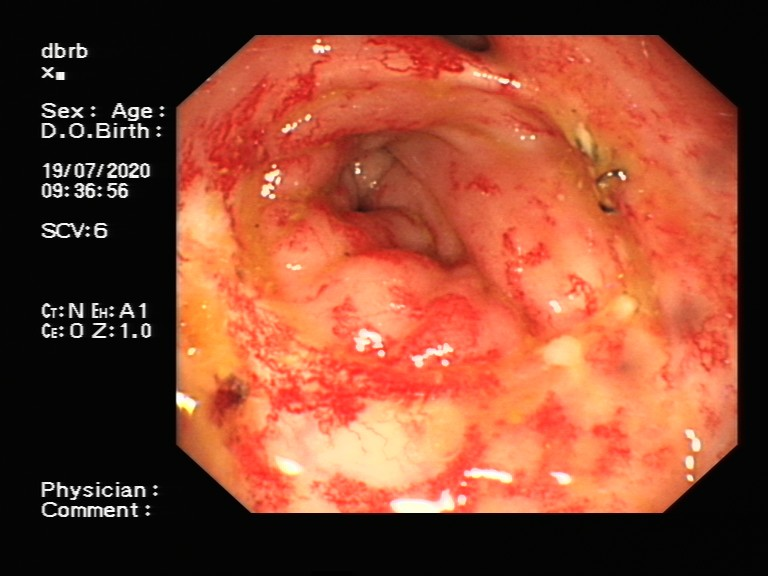
C.
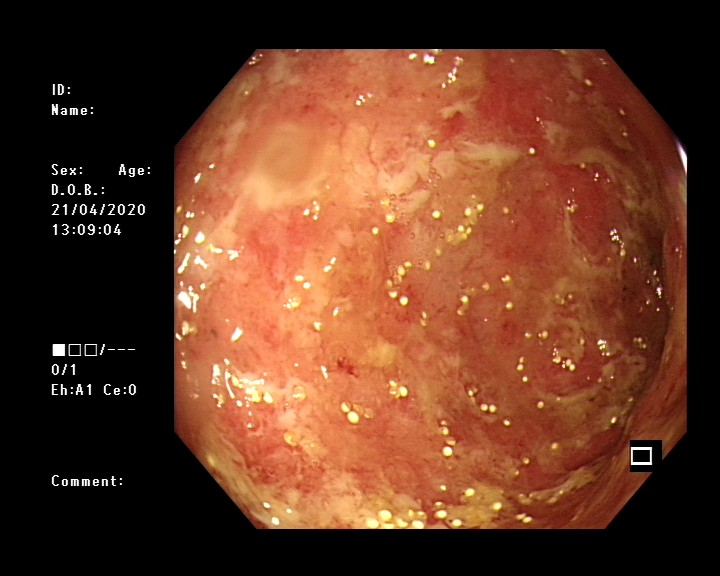
D.
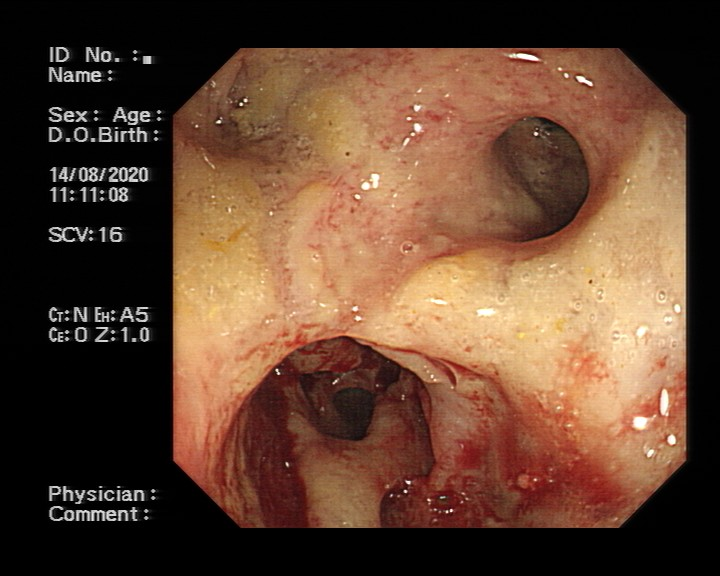


**48.Which is the contraindication of colonoscopy?()**

A. Lower gastrointestinal bleeding of unknown origin

B. Diarrhea, constipation, stool habits change

C. Severe active colitis

D. Colonic polyps or early cancers should be resected and treated under endoscope

**49. The following figure can be diagnosed as ()**


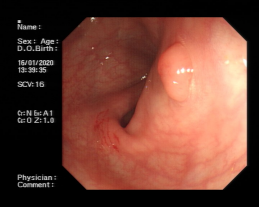


1. Colonic Polyp Type I B. Colonic Polyp Type II C. Colonic Polyp Type III D. Colonic Polyp Type IV

**50. The error of cleaning the intestinal tract before colonoscopy polyps is ()**

A. Take magnesium sulfate

B. Take mannitol

C. Take compound polyethylene glycol electrolyte powder

D. Take traditional Chinese medicine to guide diarrhea
