# Supplementary material for: A novel approach to improving colonoscopy learning efficiency through a colonoscope roaming system: randomized controlled trial
Source: PeerJ Comput Sci. 2023 Jun 9;9:e1409. doi: 10.7717/peerj-cs.1409 (PMC10280502; doi:10.7717/peerj-cs.1409)
Supplement: Supplemental Information 3 [file peerj-cs-09-1409-s003.docx]

**
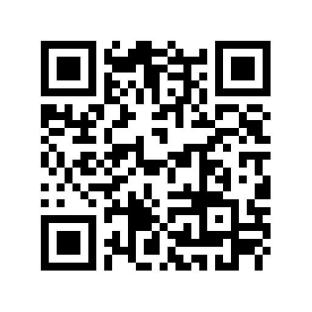
Grading Table for Colonoscopy Examination**

**Candidate number： Score：**

| Examination contents | | | Fraction | Score |
| --- | --- | --- | --- | --- |
| 1.Colonoscopy forward operation | Preparation before the examination, holding colonoscope method | | 5 |  |
|  | Operation from the anus to rectum | | 5 |  |
|  | Operation through sigmoid colon | | 5 |  |
|  | Reaching splenic flexure through descending colon | | 5 |  |
|  | Entering hepatic flexure through transverse colon | | 5 |  |
|  | Access to ascending colon | | 5 |  |
|  | Entering the ileum through the ileocecal valve | | 5 |  |
| 2.Colonoscopy withdrawal observation | Observation of the anus to rectum | | 5 |  |
|  | Observation of sigmoid colon | | 5 |  |
|  | Observation of descending colon | | 5 |  |
|  | Observation of transverse colon | | 5 |  |
|  | Observation of ascending colon | | 5 |  |
|  | Observation on ileocecal valve and appendix opening | | 5 |  |
|  | Observation of the ileum | | 5 |  |
| 3. Overall colonoscopy examination time and fluency | | | 5 |  |
| 4. Response, comfort and satisfaction of patients during colonoscopy | | | 5 |  |
| 5. Position and definition of collected image | | | 10 |  |
| 6. Diagnostic accuracy under colonoscope | | | 10 |  |
| Total | | | 100 |  |
| Errors or deficiencies of candidates: | | | | |
| Signature of assessment expert: | | Date: | | |
